# A Randomized Clinical Trial Comparing Two Treatment Strategies, Evaluating the Meaningfulness of HAM-D Rating Scale in Patients With Major Depressive Disorder

**DOI:** 10.3389/fpsyt.2022.873693

**Published:** 2022-05-27

**Authors:** Junaid Asghar, Madiha Tabasam, Maha M. Althobaiti, Amal Adnan Ashour, Mohammed A. Aleid, Osamah Ibrahim Khalaf, Theyazn H. H. Aldhyani

**Affiliations:** ^1^Faculty of Pharmacy, Gomal University, D. I. Khan, Pakistan; ^2^Department of Computer Science, Taif University, Taif, Saudi Arabia; ^3^Department of Oral & Maxillofacial Surgery, Taif University, Taif, Saudi Arabia; ^4^College of Education, King Faisal University, Al-Ahsa, Saudi Arabia; ^5^Al-Nahrain Nanorenewable Energy Research Center, Al-Nahrain University, Baghdad, Iraq; ^6^Applied College in Abqaiq, King Faisal University, Al-Ahsa, Saudi Arabia

**Keywords:** major depressive disorder, escitalopram, nortriptyline, antidepressant, HAM-D, remission, response

## Abstract

**Introduction:**

Due to the complexity of symptoms in major depressive disorder (MDD), the majority of depression scales fall short of accurately assessing a patient's progress. When selecting the most appropriate antidepressant treatment in MDD, a multidimensional scale such as the Hamilton Depression Rating scale (HAM-D) may provide clinicians with more information especially when coupled with unidimensional analysis of some key factors such as depressed mood, altered sleep, psychic and somatic anxiety and suicidal ideation etc.

**Methods:**

HAM-D measurements were carried out in patients with MDD when treated with two different therapeutic interventions. The prespecified primary efficacy variables for the study were changes in score from baseline to the end of the 12 weeks on HAM-D scale (i.e., ≤ 8 or ≥50% response). The study involved three assessment points (baseline, 6 weeks and 12 weeks).

**Results:**

Evaluation of both the absolute HAM-D scores and four factors derived from the HAM-D (depressed mood, sleep, psychic and somatic anxiety and suicidal ideation) revealed that the latter showed a greater promise in gauging the anti-depressant responses.

**Conclusion:**

The study confirms the assumption that while both drugs may improve several items on the HAM-D scale, the overall protocol may fall short of addressing the symptoms diversity in MDD and thus the analysis of factor (s) in question might be more relevant and meaningful.

## Introduction

Major depressive disorder (MDD) or unipolar depressive disorder is a syndrome most frequently diagnosed in psychiatric practice. It is characterized by low mood, loss of interest or pleasure and decreased energy, reduced self-esteem and self-confidence in usual activities and is associated with a paralyzed social status (1, 2). Around 280 million people worldwide suffer from depression. MDD is distinct from normal changes in mood and/or short-term emotional responses to everyday challenges. Each year, an estimated 5% of adults globally suffer from depression, yet it continues to be a neglected global health concern, with the majority of cases occurring in young people (3).

There is widespread recognition that this disorder is not a homogenous entity, and that further clinical characterization of the patient is required to customize the treatment plan. A range of pharmacotherapies have been demonstrated to be “equivalent” in the treatment of the syndrome in clinical trials, and these interventions are thus generally considered as interchangeable (4). Pharmacotherapy for depression is generally multifactorial and typically based on the clinician's and/or patient's preference and on tolerability issues and this could be one of the reasons why the majority of people diagnosed with depression do not achieve remission following their initial treatment (5), however, achieving complete remission of depressive symptoms and the return to normal daily functioning are the ultimate goals of antidepressant therapy. It has been demonstrated in studies that achieving remission and maintaining antidepressant therapy for a long period of time after the acute symptoms have subsided can help to prevent relapse or recurrence of the psychiatric episode and restoration of social and occupational functioning (6).

Antidepressants were first introduced to the field of psychopharmacology in the 1960s, and since then the 17-item Hamilton Depression Rating Scale (HAM-D17 or HDR) has become one of the most widely used scales to quantify the severity of symptoms of depression and determine the treatment responses. Response size is a widely used variable in antidepressant clinical trials (7) and it is usually defined as a score reduction of 50% or more on standardized depression scales. HAM-D is still considered the “gold standard” in determining the efficacy of antidepressant treatments (8) however, according to some studies, its score does not appear to be a sufficient measure of the antidepressant outcome during a clinical trial. Because the HAM-D is not a unidimensional scale (9). When developing an antidepressant treatment strategy, a more targeted approach should be used to describe the antidepressant profiles of different therapeutic agents, such as focusing on the individual item scoring, for example, changes in sleep, suicidal behavior, psychosomatic factors, appetite, or weight loss.

The studies show that a depressed patient who responds with a 50% reduction in the HAM-D score may still experience significant symptoms especially if the patient was severely depressed prior to the initiation of therapy. Remission is defined according to post-treatment scores of a depression rating scale and is commonly defined as a low absolute score of ≤7 on the HDR (10, 11). However, response does not always imply remission (12).

Many antidepressants, such as SSRIs, have been widely used to treat depressive symptoms, but they have been shown to disrupt sleep and cause other negative effects such as suicidal thoughts and changes in appetite, whereas others with sedative properties (e.g., TCAs) improve sleep, but may cause problems over time due to oversedation. As a result, patients on various antidepressants complain about treatment failure. Due to the activation of 5-HT2 receptors and an increase in noradrenergic and dopaminergic neurotransmission, some antidepressants have been shown to impair sleep quality. Among them, most prominent are selective serotonin reuptake inhibitors (SSRI), serotonin and norepinephrine reuptake inhibitors (SNRI), norepinephrine reuptake inhibitors (NRI), monoamine oxidase inhibitors (MAOI), and tricyclic antidepressants (TCA) (13).

## Methods

### Participants

Newly diagnosed community-dwelling outpatients (*n* = 500) with MDD on initial treatment attempt, aged 20–50 years of either gender, living in D.I.Khan city, KPK province of Pakistan were enrolled in the present study. The benefits and potential risks of study participation were fully explained to each patient. All participants met the defined eligibility criteria and gave informed consent for the data collection. Baseline psychiatric and somatic symptoms related to MDD, and the medications' response were evaluated at each visit.

### Inclusion Criteria

Patients were included in this trial when they (i) were awaiting to be treated with routine mental health care; (ii) were 20-50 years; (iii) met criteria of a major depressive episode (according to DSM-V); (iv) and who returned the signed informed consent form.

### Exclusion Criteria

Patients were excluded in case of (i) presence of any acute or unstable medical condition; (ii) concomitant use of any psychotropic drug; (iii) patients with a history of substance abuse (iv) pregnant and lactating mothers; (iv) patients with multiple disorders e.g., bipolar disorder, obsessive-compulsive disorder (OCD), post-traumatic stress disorder (PTSD) and eating disorders; thyroid dysfunction (v) and terminally ill patients.

### Drugs Used

#### Escitalopram

It is the active enantiomer of citalopram and belongs to the SSRIs (selective serotonin reuptake inhibitors) class. Other members in this therapeutic category include fluoxetine, paroxetine and sertraline and are currently the most widely used antidepressants. Escitalopram has been approved as a first line treatment option for major depressive disorder and generalized anxiety disorder (GAD). It increases the extracellular level of serotonin by inhibiting its reabsorption into the presynaptic cell, thereby increasing the level of serotonin available to bind to the postsynaptic receptor in the synaptic cleft (14, 15).

#### Nortriptyline

It belongs to the tricyclic antidepressants (TCAs) category. These drugs have historically played a significant role in the pharmacotherapy of MDD and are still used as a first line option. Nowadays, other antidepressant agents such as SSRIs and serotonin-noradrenaline reuptake inhibitors (SNRIs) are frequently considered as first line in the treatment of MDD. TCAs are still prescribed in cases of poor tolerability and/or a high rate of non-response to SSRIs and SNRIs (16, 17). The majority of TCAs work as SNRIs by inhibiting the serotonin transporter (SERT) and the norepinephrine transporter (NET), resulting in an increase in synaptic concentrations of these neurotransmitters and hence improved neurotransmission (18). The World Health Organization (WHO) and the World Federation of Societies of Biological Psychiatry (WFSBP) guidelines indicate that TCAs, SSRIs, SNRIs, and the newer antidepressants; mirtazapine and bupropion have no general preference (19, 20).

### Study Design and Randomization

**Figure 1:**
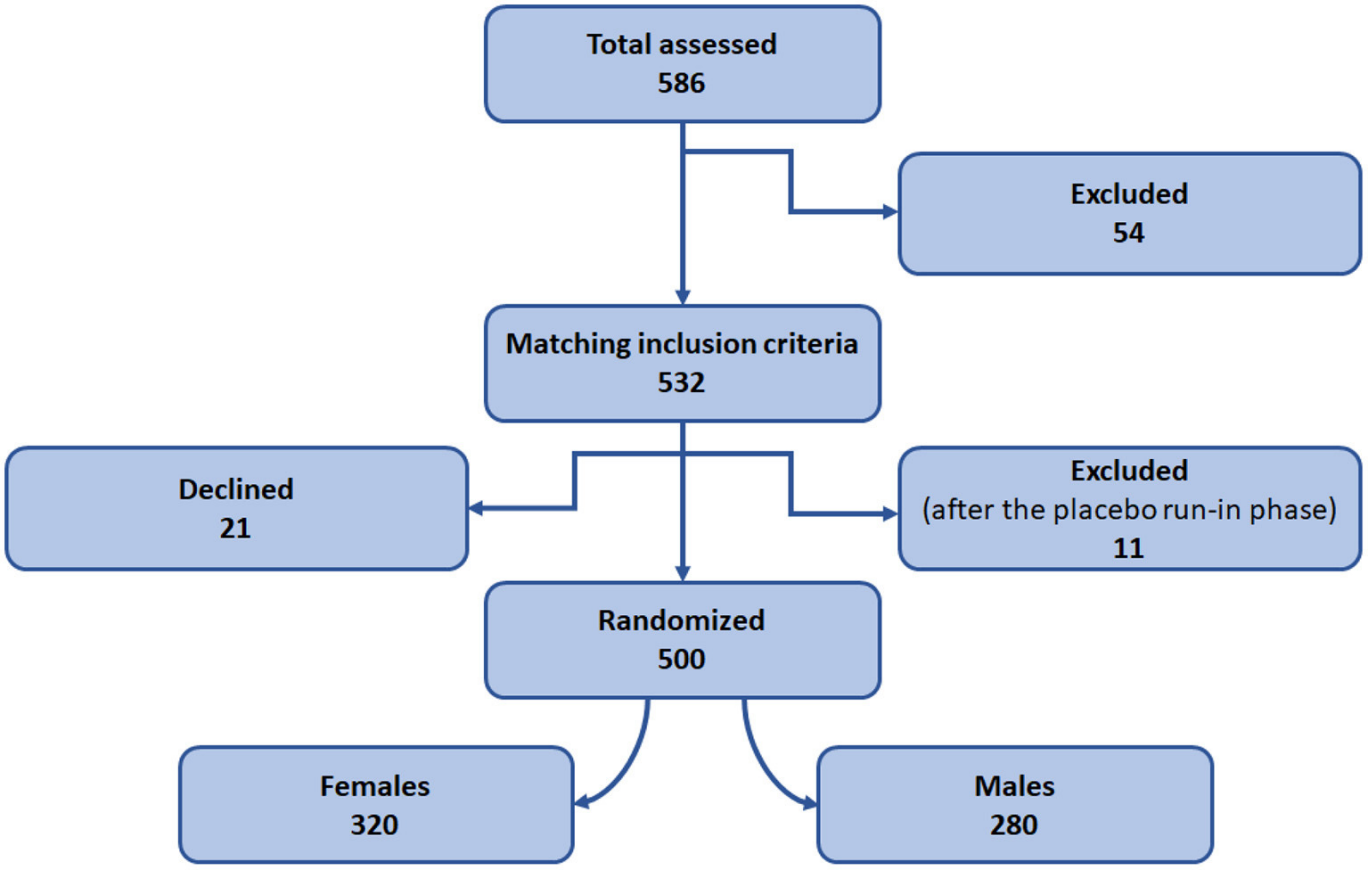
Study design and patient recruitment. Diagram representing design of the randomized control trial.

It is an open-label, randomized, fixed-dose, parallel-design study conducted at the psychiatric OPD, DHQ/TH, MTI, D.I.Khan. The patients were recruited and randomized into the study as detailed in the Figures 1, 3. A placebo run-in phase was performed in the post-inclusion/ pre-randomization period in which all the patients were given a placebo for a period of 2 weeks and the patients were assessed, and anyone who had improved substantially was excluded from the study. The investigator who conducted the randomization was not engaged in the medication dispensing, patient inclusion, or follow-up. The patients were randomized to receive either escitalopram 10 mg/day or nortriptyline 25 mg/day. The drug dosages were determined by the investigators' clinical judgment, taking into account the newly diagnosed participants' response and tolerability. The baseline data were collected, and the patients were examined at 6 weeks interval for drug responses. Overall, the data were collected at three time points (0, 6, and 12 weeks).

Psychiatric nurses monitored and ensured the drug adherence. DSM-V criteria (HAMD-17) was used to evaluate the total scores and subscore variables pre and post treatment. The answers were scored from 0 to 2 or 0 to 4 and summed up to obtain an overall score, according to the HAM-D protocol. Out of 17 items, nine items were sub scored from 0 to 2 while eight items were sub scored from 0 to 4, in which 0 represents symptoms “not present” while 4 means “severe” symptoms.

A score of 8 or less was considered equivalent to a remission. Clinical efficacy was defined as 50% or greater reduction in HAM-D rating scores, indicating a positive treatment response. Partial response was defined as an improvement between 25 and 49%. The primary efficacy parameter was measured as the mean change of scores from baseline to end of treatment between escitalopram and nortriptyline treated groups.

### Aims

To assess the usefulness and robustness of the HAM-D scale (absolute and individual factors scores) in a two-prong approach, comparing the efficacies of escitalopram (selective serotonin reuptake inhibitor) and nortriptyline (tricyclic antidepressant), particularly targeting the sensitivity of psychiatric and somatic subscales in diagnosing patients with MDD. The prespecified primary efficacy variables for the study were changes in score from baseline to the end of the 12 weeks on HAM-D scale (i.e., ≤8 or ≥50% reduction in HAMD-17 score from baseline to endpoint).

### Data Analyses

The effect size was calculated for the difference in mean change percent for each category. The data is presented as mean ± standard error mean and the p-value threshold of ≤ 0.05 is considered as significant. Changes in the HAMD-17 absolute scores and subscores from the baseline to endpoints were analyzed using one-way/ or two-way ANOVA. The *post-hoc* analysis included Dunnett's and/or Tukey's tests.

## Results

### Baseline Characteristics

Clinical characteristics at baseline were assessed for all the patients (*n* = 500) using Clinical Global Impressions (CGI) Scale to ascertain patients' symptoms severity profile, prior to the initiation of pharmacotherapy. Patients were evaluated by a panel of psychiatrists and the CGI-S responses were analyzed on a 7-point scale ranging from 1 = not ill, to 7 = extremely ill as shown in the Table 1.

**Table 1:** Patients' clinical characteristics.

**Clinical characteristics**	***n*** **= 500**
**CGI-S score**	***n*** **(%)**
1- Normal	0 (0.0 %)
2- borderline ill	20 (4%)
3- Mildly ill	23 (4.6 %)
4- Moderately ill	230 (46 %)
5- Markedly ill	196 (39.2 %)
6- Severely ill	31 (6.2 %)
7- Among the most extremely ill patients	0 (0.0 %)

Following that, five treatment outcomes were evaluated over a 12-week period (i) overall comparative efficacy of the two antidepressants; (ii) gender-based treatment response; (iii) age-based treatment response; (iv) and efficacy in treating psychosomatic disorder.

### Overall Comparative Efficacy

**Figure 2:**
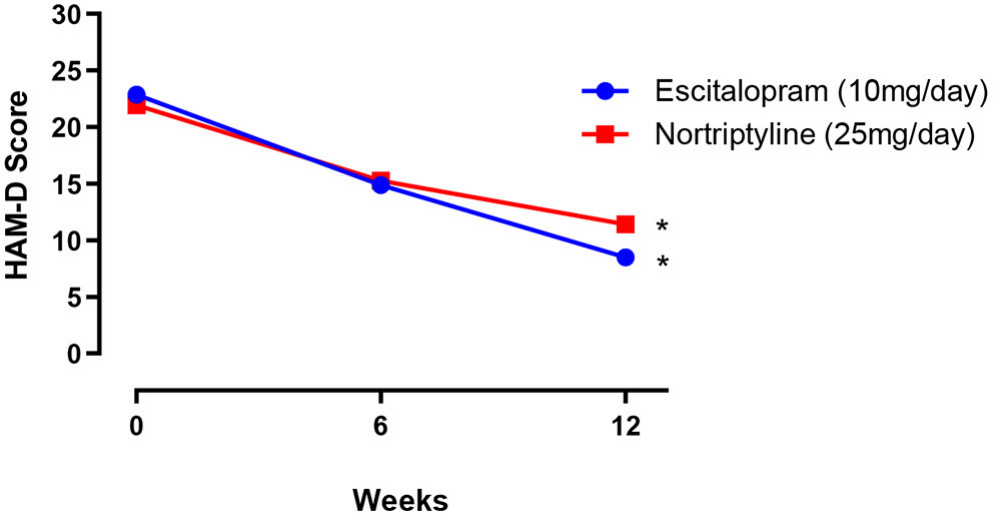
The mean ± SEM on HAM-D depression scale (*n* = 250 in each group), showing baseline and post-treatment scores at 6 and 12 weeks in patients randomly allocated to two drugs: either escitalopram or nortriptyline. *p < 0.05 shows the significant improvement in baseline depressive symptoms (one-way ANOVA).

Both male and female patients were randomly divided into two treatment groups of equal size (250 patients in each group) either on escitalopram (10 mg/day) or nortriptyline (25 mg/day) monotherapy, administered over a period of 12 weeks. In the first group, patients with depressive symptoms (baseline 22.9 ± 0.7) were given escitalopram (10 mg/day) over a period of 12 weeks, which resulted in a significant reduction of symptoms (8.50 ± 0.5) and a clinical response was demonstrated (62.9%) at the end of the treatment plan. Whereas, patients on nortriptyline (25 mg/day), showed a partial improvement (47.9%). Clinical response/ efficacy was achieved only in terms of ≥50% reduction in baseline HAM-D) in the escitalopram group (Figure 2; Table 2).

**Table 2:** The data show the mean ± SEM on 17- item HAM-D depression scale (*n* = 250 in each treatment group), compared to baseline at 6 and 12-weeks post-treatment scores.

**Weeks**	**Escitalopram Mean ± SEM**	**Improvement (%)**	**Nortriptyline Mean ± SEM**	**Improvement (%)**
0	22.9 ± 0.6	-	21.9 ± 0.6	-
6	14.9 ± 0.8	34.9	15.2^*^ ± 0.6	30.6
12	8.5 ± 0.5	62.9^*^	11.4 ± 0.8	47.9

*The patients were kept on two drugs: escitalopram and nortriptyline (monotherapy) for up to 12 weeks and the percent improvement of symptoms was recorded for each drug group*.

* *Clinical response/remission was defined as ≤ 8 or ≥50% reduction in baseline HAM-D*.

### Gender Based Treatment Response

**Figure 3:**
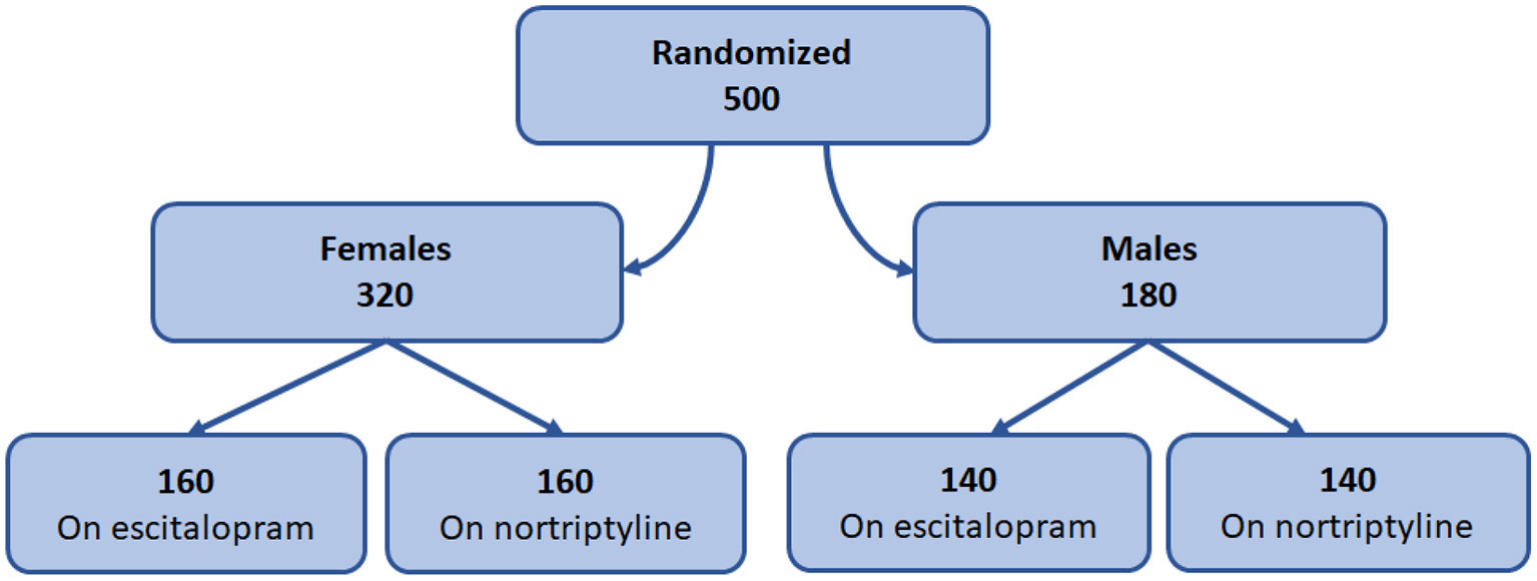
Gender-based treatment protocol. Diagram representing the design of gender-based randomization and treatment plan.

Of the 500 patients enrolled in the study, 180 (36%) were males and 320 (64%) females. Although the number of male and female patients recruited were different, we avoided the block randomization (21) and the imbalance in the number was kept the same to prevent the selection bias (22). All the patients were randomly allocated to one of the two treatment groups as shown in the Figure 3.

**Figure 4:**
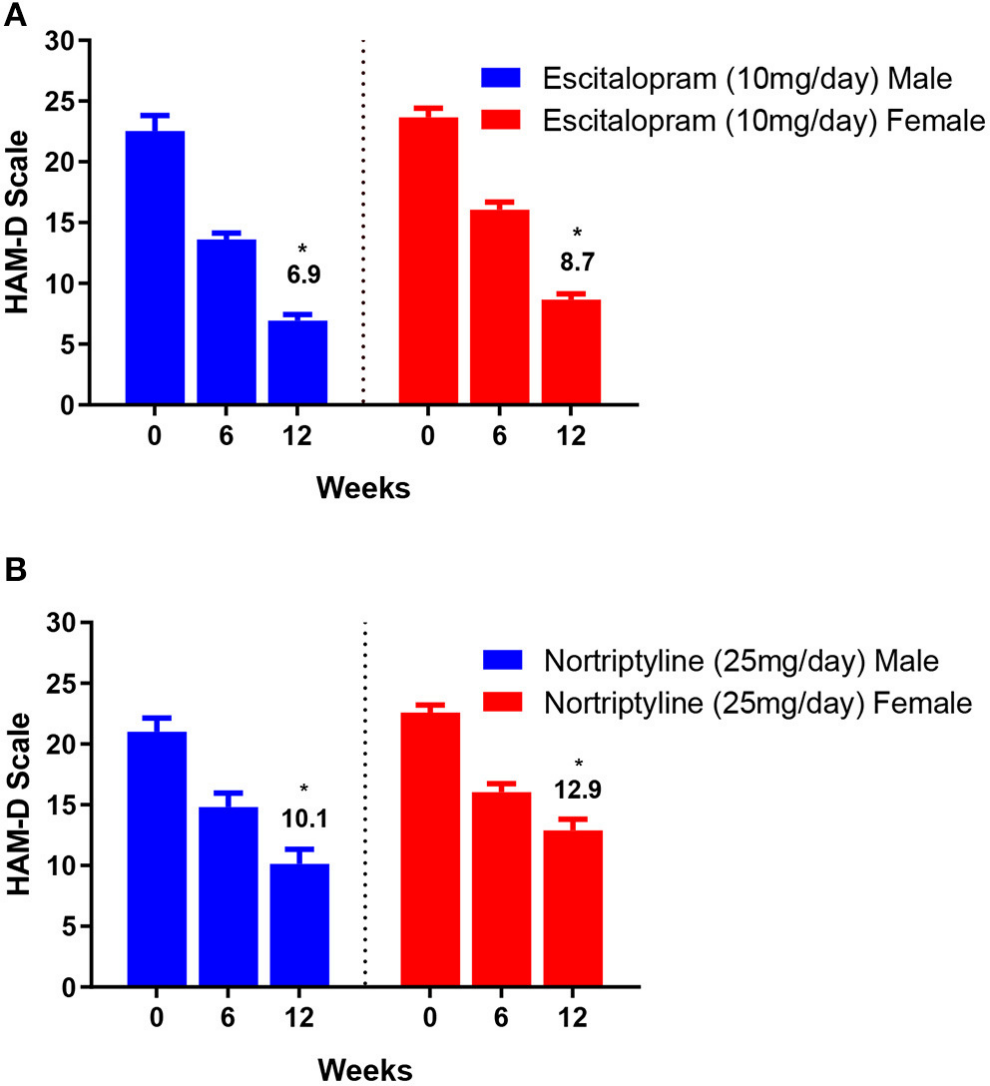
**(A,B)** Show the gender-based data on 17- item HAM-D depression scale in patients assigned to two different treatment modalities, i.e., escitalopram and nortriptyline for up to 12 weeks. *p < 0.05 shows the significant improvement in baseline depressive symptoms (one-way ANOVA).

The change in total mean score (from baseline to endpoint) was evaluated for both the groups. At the end of the therapy, improvement in depressive symptoms was associated with a decrease of −6.9 and −8.7 points on escitalopram in male and female patients, respectively, whereas, nortriptyline treatment resulted in an average reduction of −10.1 and −12.9 within male and female patients, respectively (Figures 4A,B; Table 3). In the male group, a significant clinical response was achieved on escitalopram and nortriptyline-treated patients (69.3 and 51.9%, respectively) at 12 weeks. However, in the female group, only escitalopram was significantly more effective (63.1%) than nortriptyline which demonstrated only partial response (42.9%) as shown in the Table 3.

**Table 3:** The gender-based data on 17- item HAM-D depression scale.

**Drugs**	**Weeks**	**Male patients** **(Mean ± SEM)**	**Improvement (%)**	**Female patients** **(Mean ± SEM)**	**Improvement (%)**
Escitalopram	0	22.5 ± 1.3	-	23.7 ± 0.8	-
	6	13.6 ± 0.5	39.6	16.1 ± 0.6	32.1
	12	6.9^*^ ± 0.5	69.3^*^	8.7 ± 0.7	63.1^*^
Nortriptyline	0	21.0 ± 1.1	-	22.6 ± 0.6	-
	6	14.8 ± 1.2	29.5	16.0 ± 0.7	29.2
	12	10.1 ± 1.2	51.9^*^	12.9 ± 0.9	42.9

*Both male and female patients were randomly assigned to escitalopram and nortriptyline for up to 12 weeks. Mean ± SEM and percent improvement of symptoms were recorded for each drug group*.

* *Clinical response/remission was defined as ≤ 8 or ≥50% reduction in baseline HAM-D*.

### Age-Based Treatment Response

**Figure 5:**
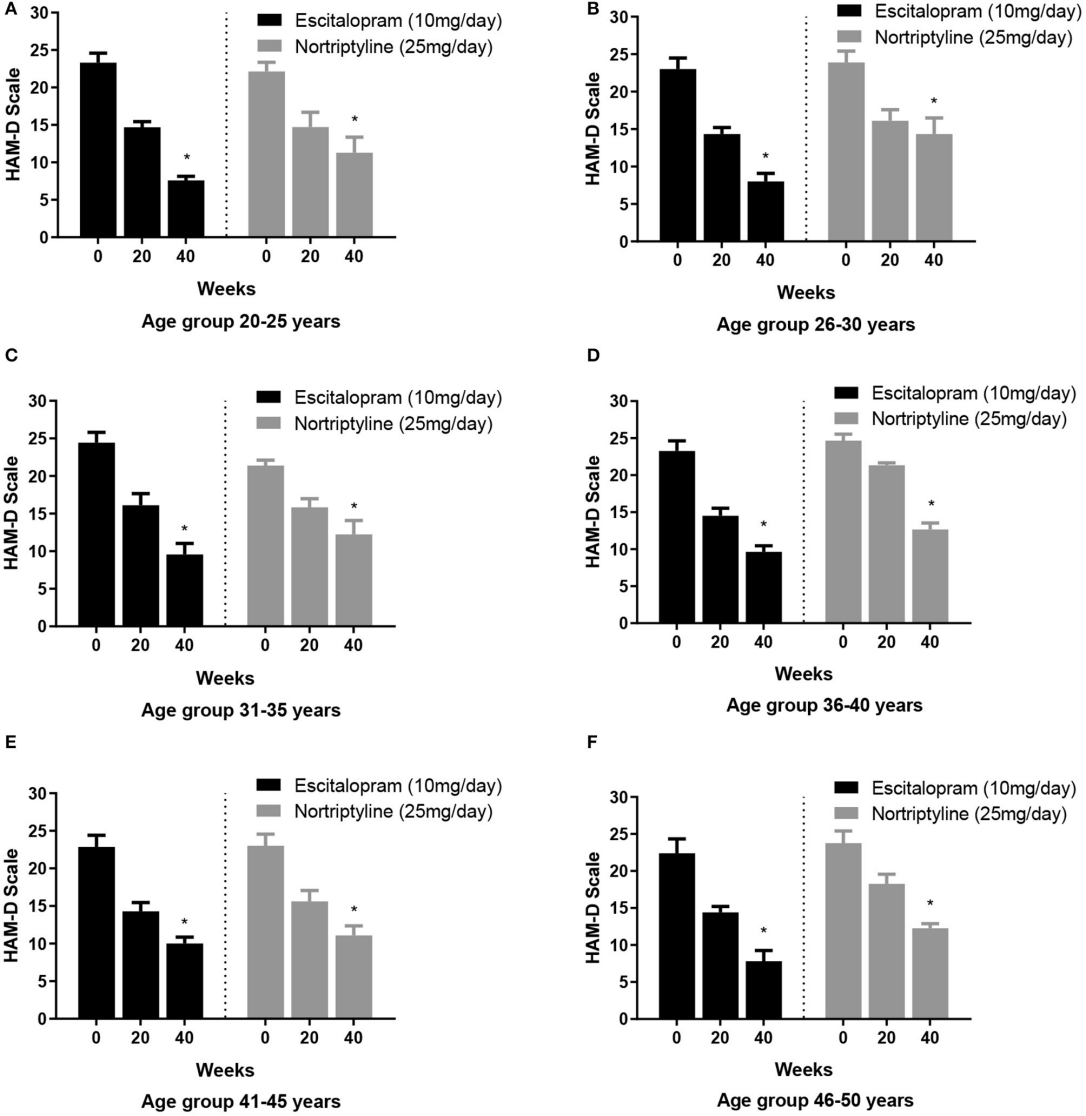
**(A–F)** Show the age group-based data on 17- item HAM-D depression scale. Participants in different age groups (15–20, 21–25, 26–30, 31–35, 36–40, 41–45, and 46–50 years) were randomly allocated to escitalopram and nortriptyline for up to 6 weeks. *p < 0.05 shows the significant improvement in baseline depressive symptoms (one-way ANOVA).

To test whether escitalopram or nortriptyline might differ in efficacy to minimize anxiety/ somatization sub-scores in different age groups, an aged-based comparison was performed. The patients of either sex were divided into 6 age groups: (20–25, 26–30, 31–35, 36–40, 41–45, and 46–50 years) and were randomly allocated to either escitalopram. Both the drugs resulted in significant reduction of symptoms on HAM-D rating scale and produced a statistically significant response in all the age group at the end of the treatment plan (^*^p < 0.05; One-way ANOVA) (Figures 5A–F). However, a varied clinically response was achieved across different age groups as summarized in Table 4.

**Table 4:** The age group-based data on 17- item HAM-D depression scale.

**Age group**	**Weeks**	**Escitalopram group** **Mean ±SEM**	**Improvement (%)**	**Nortriptyline group** **Mean ±SEM**	**Improvement (%)**
20–25	0	23.3 ± 1.3	-	22.5 ± 1.2	-
	6	14.7 ± 0.7	36.9	14.7 ± 1.9	33.6
	12	7.6^*^ ± 0.5	67.4^*^	11.3 ± 2.07	48.9
26–30	0	23.0 ± 1.5	-	23.9 ± 1.5	-
	6	14.3 ± 0.9	37.8	16.1 ± 1.5	32.6
	12	8.0^*^ ± 1.09	65.2^*^	14.3 ± 2.2	40.2
31–35	0	24.4 ± 1.4	-	21.4 ± 0.7	-
	6	16.1 ± 1.5	34.0	15.8 ± 1.1	26.2
	12	9.6 ± 1.5	60.7^*^	12.2 ± 1.9	42.9
36–40	0	23.3 ± 1.4	-	24.6 ± 0.8	-
	6	14.5 ± 1.0	37.8	21.3 ± 0.3	13.4
	12	9.6 ± 0.8	58.8^*^	12.6 ± 0.8	48.8
41–45	0	22.9 ± 1.6	-	23.0 ± 1.6	-
	6	14.3 ± 1.2	37.6	15.6 ± 1.5	32.2
	12	10.0 ± 0.9	56.3^*^	11.1 ± 1.3	51.7^*^
46–50	0	22.4 ± 1.9	-	23.8 ± 1.6	-
	6	14.4 ± 0.8	35.7	18.3 ± 1.3	23.1
	12	9.8 ± 1.5	56.3^*^	11.5 ± 0.6	51.7^*^

*Participants in different age groups (20–25, 26–30, 31–35, 36–40, 41–45, and 46–50 years) were randomly allocated to escitalopram and nortriptyline for up to 12 weeks. Mean ± SEM and percent improvement of symptoms were recorded for each drug group*.

* *Clinical response/ remission was defined as ≤ 8 or ≥50% reduction in baseline HAM-D*.

### Psychosomatic Disorder and Treatment Response

Some of the HAMD-17 data [depressed mood, psychic anxiety, somatic anxiety symptoms (indigestion, palpitations and headache) and insomnia (initial and middle)] from 500 patients of the 12-week trial comparing the effectiveness of escitalopram and nortriptyline were converted to subscale scores and analyzed during the antidepressant treatment course.

**Figure 6:**
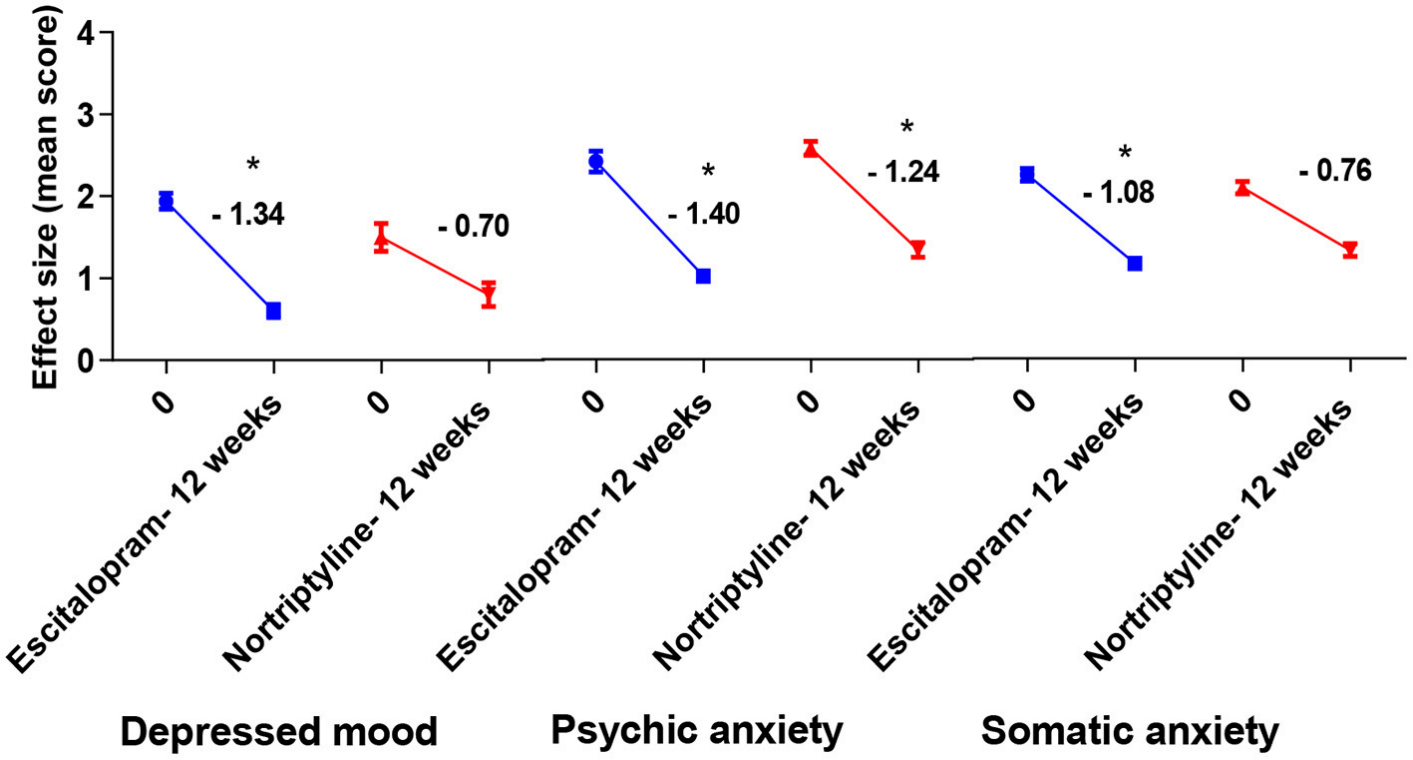
Plots show the mean changes/ reduction in effect size from the baseline for psychiatric symptoms (depressed mood and psychic anxiety) and somatic anxiety symptoms (indigestion, palpitations and headache) on the HAM-D scale. *p < 0.05 shows significant improvement (one-way ANOVA, followed by Dunnett's multiple compassion test).

**Figure 7:**
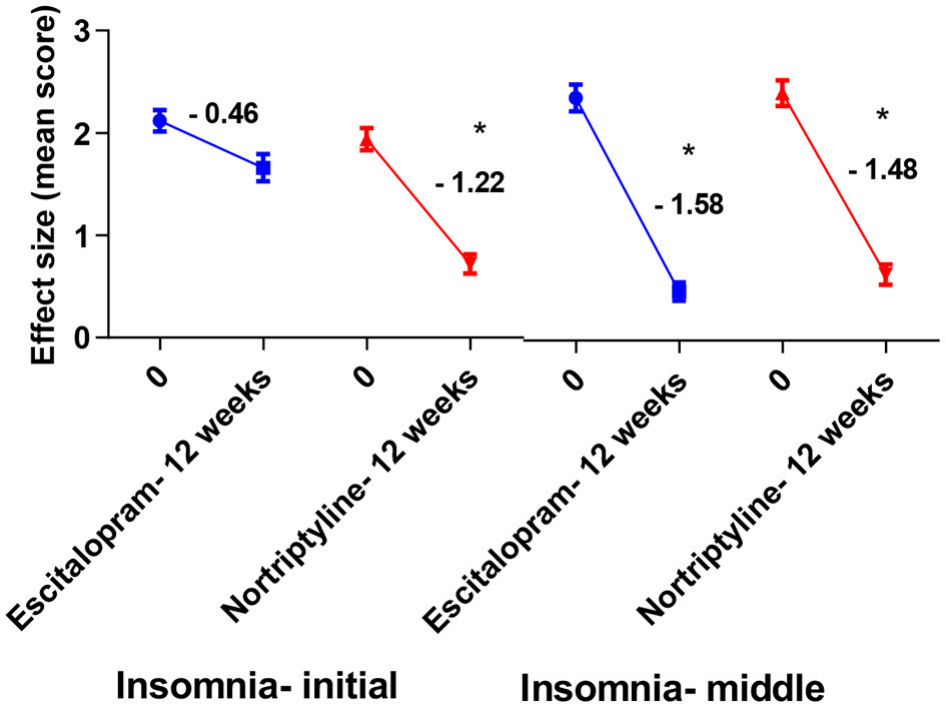
Plots show the mean changes/ reduction in effect size from the baseline for insomnia (initial and middle) on the HAM-D scale. *p < 0.05 shows significant improvement (one-way ANOVA, followed by Tukey's multiple compassion test).

**Figure 8:**
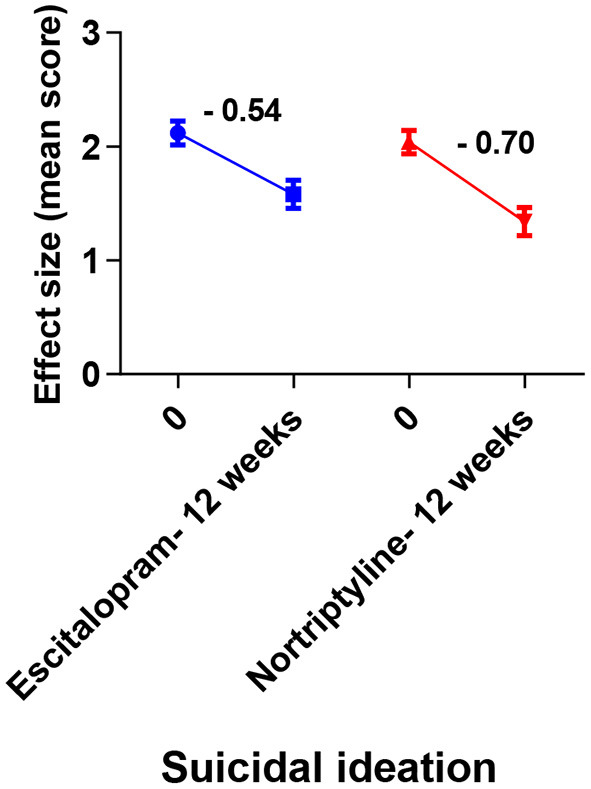
Plots show the mean changes/ reduction in effect size from the baseline for suicidal ideation on the HAM-D scale. No significant change was observed across different data sets (one-way ANOVA, followed by Tukey's multiple compassion test).

A standard effect size analysis showed improvement in psychosomatic symptoms, following up to 12 weeks of therapy with either escitalopram or nortriptyline monotherapy. Analysis of subscale scores for anxious depression such as depressed mood (sadness, worthlessness, tendency to weep) and psychic anxiety (chronic excessive worry) were assessed. Additionally, analysis of the sub-scores such as insomnia (initial and middle) and somatic anxiety (indigestion, palpitations and headache) were carried out to assess if there was any improvement in the baseline severity. The changes in various psychosomatic parameters/subscale scores are shown in Figures 6–8 and summarized in Table 5.

**Table 5:** The comparison of mean changes in effect size compared to baseline for depressed mood, psychic anxiety, somatic anxiety and insomnia and suicidal ideation (subscale scores) on the HAM-D scale at 12 weeks among patients with MDD, treated with escitalopram and nortriptyline.

**Drug**	**Change in effect size (difference of mean scores)**					
	**Depressed mood**	**Psychic anxiety**	**Somatic anxiety**	**Insomnia**		**Suicidal ideation**
				**Initial**	**Middle**	
Escitalopram	−1.34^*^ ± 0.1	−1.40^*^ ± 0.1	−0.94 ± 0.1	−0.46 ± 0.1	−1.58^*^ ± 0.1	0.54 ± 0.1
Nortriptyline	−0.70 ± 0.2	−1.24^*^ ± 0.1	−1.2^*^ ± 0.1	−1.22^*^ ± 0.1	−1.48^*^ ± 0.1	0.70 ± 0.2

* *p < 0.05 shows significant improvement (one-way ANOVA, followed by Tukey;s multiple compassion test)*.

## Discussion

The aim of this research was to evaluate the HAM-D scale's suitability and practicability when comparing two different treatment outcomes in a group of patients who were treated according to the general protocols in a hospital setting. MDD usually goes under-treated as the patients do not respond equally to the available antidepressant choices due to multiple factors such as complexities in psychosocial variables, lack of proper assessment, poor medication response and lack of adherence to the treatment protocols. Consequently, the overall aim of the project was to evaluate the usefulness of HAM-D scale and, followed by a micro-analytic approach derived from HAM-D, in which four specific items were analyzed separately.

We selected and analyzed the data on the basis of a set of primary efficacy variables on HAM-D from baseline to the end of 12 weeks (i.e., ≤ 8 or ≥50%). According to outcomes of a meta-analysis on MDD and different antidepressants, about one-fourth of the studies showed remission within 12 weeks, one-third of the studies showed remission within 6 months, while one and a half studies showed remission within the period of 12 months (23). A cohort study conducted in primary health care showed the highest remission rate of depressive features in the third and 6 months of the study (24). Antidepressants reach a plateau or stable effect after 6–12 weeks of treatment (25); therefore, a 12-week study was conducted in order to examine the full range of therapeutic efficacies to antidepressants.

The HAMD-17 item scale has been a widely used tool in psychiatric research to assess the severity of depression. Its original version contained 17 items, but it kept updating and its latest revision took place in 1980. The Beck Depression Inventory (BDI) is another widely used depression scoring tool in research that has evolved over time; its most recent version, known as the BDI-II, was introduced in 1996. One of the limitations of these scales is that the side effects of antidepressants could intensify the item scores on these scales and thus mask the true positive effects of the antidepressant agents (26, 27).

Both escitalopram and nortriptyline are the frequently used antidepressant agents in treating MDD. In this study, we used a fixed dose of escitalopram, 10 mg/day and nortriptyline, 25mg/day in our newly diagnosed MDD patients. Numerous placebo-controlled trials have shown that when patients with MDD received escitalopram at a dose of 10 mg/day, it had a significantly greater efficacy than placebo. Furthermore, the escitalopram group had a higher rate of remission than the placebo group. Consequently, 10 mg/day was found to be safe and effective in the initial stages of the MDD. In terms of reduction in depression scores, the efficacy was greater with escitalopram than with placebo at the first or second week and were maintained throughout treatment at these doses (28, 29). Studies reveal that TCAs initial and maintenance dosages are determined empirically and are not substantiated by strong clinical evidence. Lower doses of nortriptyline (25–100 mg/day) were found to be equally efficacious as higher doses with lesser adverse effect events in one review (30).

In our findings, overall comparative efficacy/ target remission (≤8 or ≥50% reduction in baseline HAM-D) of the two drugs revealed that escitalopram is significantly more effective (62.9%) in comparison to nortriptyline (47.9%). None of the drugs could achieve the other efficacy target i.e., ≤ 8 score (Table 2). As a general trend, subjects of all age groups, receiving escitalopram showed highest remission rates than nortriptyline at the end of the therapy. Furthermore, no significant difference was recorded in terms of antidepressant efficacy (absolute HAMD-17 score of ≤ 8) after 6 weeks of therapy with either drug in all the age groups. Escitalopram offered superior control of depressive symptoms and led to clinical remission at the end of the study (12 weeks) in all age groups with a reduction of 50% or more of the HAMD-17 score, however, in terms of cut-off value on HAM-D scale (≤8), only the age group 26–30 achieved the target score. On the other hand, some interesting data were obtained with nortriptyline which produced a clinical response (≥50% reduction in baseline HAM-D) in the older age groups (41–45 and 46–50), however, it could not produce the same effect in the earlier age groups (20–40 years) (Table 5). In order to investigate this differential age-related drug response, a thorough search of the literature led to the extraction of a study where the author recommended TCAs to be more effective antidepressant agents for the acute and/or longer course of antidepressant therapy, particularly in elderly patients (31), however, TCAs are no longer preferred as first-line agents for geriatrics (above 60 years) due to their potential side effects, including postural hypotension, which can contribute to falls and fractures, cardiac conduction disorders and anticholinergic/ antihistaminic effects (32). There is a widely held assumption that individual differences underlie the variability in the association of antidepressants with depressive symptoms (i.e., response). To our knowledge, however, efforts to discover characteristics related with antidepressant response and their impacts on different age groups or gender have been largely ineffective. Nonetheless, depression appears to be a more heterogeneous condition than other psychotic states like schizophrenia, and the unexplained source of this heterogeneity may account for part of the observed variability in antidepressant treatment response in different age groups (33–36).

Depression is more prevalent in women as compared to men (37, 38). Females aged 14 to 25 years have been reported to have twice the prevalence rate of depression as compared to men (39–41); largely due to the hormonal fluctuations, whereas the prevalence rate before puberty remained the same in both genders (23, 39). To see if there were any differences in the rates of improvement based on gender, we tested escitalopram and nortriptyline and observed that the symptoms of males significantly improved by the end of treatment (12 weeks), leading to ≥50% reduction of symptoms, while in females, escitalopram was found to be more efficacious than nortriptyline, as the latter showed only 42.9% reduction at the end of the therapy (Table 3). Despite decades of research, there is still no clear consensus on whether there are sex-related efficacy differences in antidepressant treatment. For example, males had a considerably better therapeutic response to another tricyclic antidepressant, imipramine, than females. These differences in response could be caused by a multitude of variables, including body fat distribution, liver metabolic rates, hormone physiology, and brain interactions between estrogen and serotonin (40).

To achieve more relevant and robust outcomes, we additionally performed a factor-based evaluation of some key symptoms. Factors/ subscores analyses on HAM-D for MDD assessment may be more sensitive to antidepressant drug effects (41), so we looked at the sensitivity to depressive change for some key subscales [depressed mood, psychic anxiety, somatic anxiety symptoms (indigestion, palpitations and headache) and insomnia (initial and middle)] which performed better throughout the treatment course, with some subscales having advantage in detecting the treatment effects. Following up to 12 weeks of therapy with either escitalopram or nortriptyline monotherapy, a standard effect size analysis showed improvement in psychosomatic symptoms. Analyses of effect size scores (baseline to week 12) for the different treatment groups showed some interesting results. A *post-hoc* analysis of the effect sizes for each item (Figure 6; Table 5) showed considerable change in the escitalopram and nortriptyline group (e.g., psychiatric anxiety and somatic anxiety symptoms). The item “somatic anxiety” had the highest impact in the nortriptyline group. On the other hand, escitalopram significantly improved insomnia-middle, however, its effects on insomnia-initial were very small (Figure 7; Table 5) which means both the drugs resulted in increased sleep latency, however, the total sleep time was significantly improved in the escitalopram group.

According to Husain et al. (42), both escitalopram and nortriptyline were significantly effective in relieving painful physical symptoms and depression severity. Several studies reveal that the MDD associated somatic symptoms are difficult to treat with the available antidepressant choices (43–47). According to Marangell et al. (48), subjects receiving both nortriptyline and escitalopram for 2 weeks, showed 50% reduction in somatic anxiety, however, clinical response in terms of physical and depressive features was achieved subsequently. For example, on HAM-D 17 item scale, baseline data showed no significant difference in the severity of depression in subjects with or without painful somatic symptoms and regardless of somatic complains, remission rate for MDD remained 84%. Subjects with somatoform disorder reported having severe depressive episodes, which greatly affected the therapeutic outcomes and decreased the clinical response rate in totality. In the current study, we found a significant improvement in depressive and somatoform symptoms with time (12 weeks of therapy). Same has been investigated in some other studies that remission in somatic symptoms is associated with an overall remission of MDD symptoms, with the longer course of antidepressant therapy (43, 46, 49, 50).

Antidepressants' therapeutic efficacy may be hampered by side effects like insomnia, because continual insomnia can exacerbate depressive episodes and mask the true antidepressant effects of these drugs (51, 52). Previous studies show that TCAs produce significant improvement in normalizing sleep pattern when compared to SSRI, because of their anticholinergic and antihistaminic properties. At the same time, the sleep efficiency and depth are substantially reduced in depressed patients and changes in rapid eye movement (REM) are most commonly affected (13). SSRIs might be responsible for a disturbed sleep cycle (particularly difficulty falling asleep) (53) and this has been linked to the activation of 5-HT2 receptor which leads to mental activation and thus insomnia, and therefore, add up to the pre-existing burden of depressive symptoms. TCAs, however, due to their central anticholinergic and H_1_ blocking actions could improve sleep (54). Accordingly, our findings show that TCAs are significantly better at relieving insomnia than SSRIs, while patients in the latter group reported marked insomnia (Figure 7; Table 5).

MDD is commonly associated with suicidal thoughts/ ideation. More than 60 percent of people who have attempted suicide worldwide have MDD. There is a 20-fold higher risk of suicide among patients with MDD, compared to the general population (55, 56). To treat or prevent suicidal ideation and suicide attempts, antidepressants must be prescribed. According to pharmacoepidemiological studies, the number of suicides decreased as the use of antidepressants increased (57, 58). There have been reports of increased and new-onset suicidal activities since 1988s with TCAs. Also, the SSRIs have been the subject of debate for the past two decades, with a focus on their role in the treatment of depression and anxiety. Controversial results have been found in meta-analysis of randomized trials (59). Since suicidal events are so rare, Gunnell et al. (60) stated in their meta-analysis that SSRIs' effects could not be evaluated. Suicidal thoughts and behavior triggered by antidepressant drugs (primarily with SSRIs) are extremely rare (61). Restlessness and impulsiveness are all possible warning signs in the early stages of psychosis. Based on our study (HAM-D item-analysis protocol), no drug significantly reduced the suicidal thoughts, however, nortriptyline resulted in a larger score reduction as compared with escitalopram (Figure 8; Table 5). To address the issue, it is recommended that when treating depression for the first time, an appropriate combination therapy may be preferred over monotherapy. However, according to the current study's protocols, switching from nortriptyline to escitalopram resulted in better outcomes than switching from escitalopram to nortriptyline at the end of the study period (data not shown).

In this study, there were no unexpected side effects from the usage of escitalopram or nortriptyline. Escitalopram induced a modest weight increase, as expected, as well as nighttime insomnia. SSRIs have historically been associated with insomnia and poor subjective sleep quality (62). With our participants, we found the same as a general trend. As a result, patients were advised to take escitalopram during the daytime to circumvent nighttime insomnia. Nortriptyline has been a useful antidepressant, though the prevalence and severity of anticholinergic side effects is a downside. We discovered a correlation between efficacy and anticholinergic side effects such as dry mouth and/ or constipation in all the age groups in the current investigation (data not shown). However, no participant dropped out of the trial due to intolerance to these side effects. The delayed onset of antidepressant action has traditionally been an impediment to depression treatment. Antidepressants' complete therapeutic efficacy may take several weeks to manifest, leaving patients to endure prolonged episodes of depressive symptomatology (63) as was the case with this study. One of the most crucial aspects of the relationship of socioeconomic status to psychiatric health, and one of the most consistent associations in the field of psychiatric epidemiology, is the relationship of socioeconomic status to psychiatric disorders (64). With respect to sociocultural context, some of our participants were reluctant to accept that they had depression, and even whether treatment is needed at all. For some, depression was stigmatizing. Furthermore, convincing them to initiate the treatment was challenging in some cases.

### Limitations

Several limitations of our study are worth mentioning, including the participants, most of which were females, and all were Asian, thus limiting the study's generalizability to other populations. Similarly, during the administration of the questionnaire, special attention was paid to the evaluation of each element's meaning, without eliciting any significant questions or observations from the participants. The study was only limited to the effects of two drugs; several antidepressants were still very expensive at the time of the study and the participants preferred cost-effective and easily accessible options offered: escitalopram and nortriptyline. Using other anti-depressants such as paroxetine, bupropion, duloxetine and desvenlafaxine, may yield different outcomes.

## Conclusion

Using data from this clinical trial, we could conclude that the individual effect size analysis has some advantages over the HAM-D absolute scores for depression assessment because of its more focused factor-based approach of evaluating depressive symptoms pre and post treatment. The practicing psychiatrists might follow or want to consider tailoring our methods to their particular needs when comparing different antidepressants' efficacies.

## Data Availability Statement

The raw data supporting the conclusions of this article will be made available by the authors, without undue reservation.

## Ethics Statement

This study was approved by the Ethical Review Board (ERB) of the Gomal University, KP, Pakistan. The patients/participants provided their written informed consent to participate in this study.

## Author Contributions

All authors listed have made a substantial, direct, and intellectual contribution to the work and approved it for publication.

## Funding

Present research work is supported by Taif University Researchers Supporting Project number (TURSP-2020/130), Taif University, P.O Box 11099, Taif 21944, Saudi Arabia.

## Conflict of Interest

The authors declare that the research was conducted in the absence of any commercial or financial relationships that could be construed as a potential conflict of interest.

## Publisher's Note

All claims expressed in this article are solely those of the authors and do not necessarily represent those of their affiliated organizations, or those of the publisher, the editors and the reviewers. Any product that may be evaluated in this article, or claim that may be made by its manufacturer, is not guaranteed or endorsed by the publisher.
